# Factors Related to Satisfaction with Decision-making Regarding Human Papillomavirus Vaccination Behavior among Female University Students in Japan

**DOI:** 10.31662/jmaj.2022-0214

**Published:** 2023-11-16

**Authors:** Chie Koh, Kaori Watanabe, Minako Saho, Yukari Nakajima, Miho Furuyama, Kanako Yamada, Yuichirou Nakai

**Affiliations:** 1 Graduate School of Nursing, Osaka Metropolitan University, Osaka, Japan; 2 Koike Hospital, Hiroshima, Japan

**Keywords:** Human papillomavirus (HPV), HPV vaccination, Satisfaction, Decision-making, Sexuality, Female university students

## Abstract

**Introduction:**

Cervical cancer is the fourth most common cancer among women worldwide. Most cervical cancers are caused by persistent infection with human papillomavirus (HPV) acquired through sexual contact. Decision-making is the process of choosing among several options, and a better decision is one that the people engaged in the decision-making process express satisfaction with. Despite that HPV infection is associated with sexual behavior, no studies in Japan on HPV vaccination decision-making that include perspectives on sexuality exist. This study aimed to determine the factors that influence satisfaction with decision-making concerning HPV vaccination among female university students in Japan.

**Methods:**

The cross-sectional study was carried out by an anonymous self-administered questionnaire mail survey of 1988 female university students in Japan between April and July 2021. Of them, 301 agreed to participate in the survey. After the exclusion of those with missing data, the analysis included 252 (12.7%) students. We summarized descriptive statistics in terms of characteristics, satisfaction with decision-making regarding HPV vaccination, HPV vaccination behavior, knowledge, attitude about HPV vaccination, influencing factors, and perceptions and behaviors related to sexuality. Furthermore, we conducted multivariate analyses to investigate factors that influence satisfaction with decision-making regarding HPV vaccination.

**Results:**

Of the 252 participants, 102 (40.5%) were satisfied with their decisions regarding HPV vaccination. After adjustment for confounding factors, the multivariable-adjusted odds ratios (95% confidence intervals) for factors associated with satisfaction in decision-making regarding HPV vaccination were as follows: being vaccinated (vs. non-vaccinated) 5.46 (2.51-11.89), having high knowledge scores (vs. per 1 point) 1.09 (1.01-1.17), and having awareness about the risk of contracting sexually transmitted infections (STIs) via sexual intercourse (vs. per 1 point) 0.83 (0.72-0.96).

**Conclusions:**

Being vaccinated, having higher knowledge scores, and having lower awareness regarding the risk of STIs were associated with satisfied decision-making concerning HPV vaccination. Providing younger people with correct information about cervical cancer, HPV vaccines, and STI prevention contributes to increased satisfaction with their HPV vaccination decisions.

## Introduction

Cervical cancer is the fourth most common cancer among women worldwide ^(1)^. Most cervical cancers are caused by persistent infection with human papillomavirus (HPV) acquired through sexual contact, and most people are infected with HPV shortly after the onset of sexual activity ^(1), (2)^. HPV vaccines work best if administered before HPV exposure. For this reason, vaccination before the initiation of sexual intercourse is recommended globally, and the World Health Organization (WHO) also recommends vaccinating girls aged 9-14 years, when most have not started sexual activity ^(1)^.

The HPV vaccine was approved by the Japanese government in October 2009 and became available for use at medical institutions in December of the same year. Moreover, in April 2013, public funding was provided for HPV vaccines, targeting the population from the sixth grade of elementary school to the first grade of high school (approximately ages 11-16) as a project to promote emergency measures for vaccination against cervical cancer ^(3)^. Nevertheless, because of the issue of adverse reactions, the Japanese government withheld vaccination recommendations after only 3 months of this program, and there was no active recommendation made from June 2013 to March 2022 ^(3), (4), (5)^. Japanese media reported these adverse reactions sensationally, resulting in widespread misinformation and misunderstanding of the HPV vaccine, and distrust of the HPV vaccine persists among most Japanese people ^(6)^. As a result, HPV vaccination rates remain low in Japan, reported to be 1.9% in 2019 ^(4)^.

Decision-making is the process of choosing among several options, and a better decision is one that the people engaged in the decision-making process express satisfaction with ^(7)^. Previous studies have shown that the HPV vaccination group has a higher rate of satisfaction with decision-making ^(8)^ and with sexual behavior. There have been conflicting reports regarding the association between HPV vaccination and an earlier age of first sexual intercourse and the number of sex partners, with some studies ^(8), (9)^ that state that there is an association and others that report that there is no association ^(10)^. Unlike other diseases that cause airborne or droplet transmission, the risk of HPV infection in Japan varies greatly depending on the nature of sexual activity. Sexual activity is the most private domain of each individual, and vaccination decisions should be made after careful consideration based on one’s own values about sex. The sexual intercourse experience rate of female students in Japan (2019) has been reported to be 19.3% for high-school students and 36.7% for university students ^(11)^. In this way, university students become more familiar with the issue of sexuality by becoming more sexually active as indicated by these higher rates. Despite HPV infection being associated with sexual behavior, no studies of HPV vaccination decision-making in Japan include perspectives on sexuality such as sexual intercourse experience or awareness of the risks of getting pregnant or contracting STIs.

Clarifying the factors that are related to satisfaction with decision-making regarding HPV vaccination among female university students in Japan could be useful for determining appropriate interventions to enhance satisfaction with these decisions. Although previous studies have shown that knowledge about HPV and HPV vaccination, as well as attitudes toward HPV vaccination ^(12)^, are related to decision-making regarding HPV vaccination, the association between perspectives on sexuality and satisfaction with decision-making in terms of HPV vaccination has not yet been assessed. Thus, this study aimed to determine the factors that influence HPV vaccination behaviors, specifically including perspectives on sexuality, and how they relate to satisfaction with decision-making regarding HPV vaccination among female university students in Japan.

## Materials and Methods

### Study participants and procedure

This anonymous self-administered questionnaire mail survey was conducted from April to July 2021. The targeted list of National Councils of Nursing universities in Japan was randomly ranked, and a request for research permission was sent to the deans of those universities. The universities that gave permission for this research were selected as the research target universities. Requests were repeated until the number of research participants reached approximately 1,000 students. Non-nursing universities were randomly ranked from the list of universities on the Ministry of Education, Culture, Sports, Science and Technology website and selected in order from the top of the list. In doing so, medical-related programs (medical, pharmacy, and others) were excluded. Requests for research permission were sent to the deans of the faculties of each university, and approximately 1,000 research participants were selected via the same method as from nursing universities. Finally, we distributed anonymous self-administered questionnaires to 1,089 nursing and 899 non-nursing university female students (1,988 students overall). Of these, 301 female university students agreed to participate in the mail survey. We excluded those with missing data. A total of 252 (12.7%) female university students were included in the analysis.

The study protocol was written following the Declaration of Helsinki and was approved by the Institutional Review Boards of Osaka Prefecture University (date of approval: March 25, 2021; approval code: 2020-51). Informed consent was obtained from all participants who were included in the study.

### Outcome

Study participants were classified into two groups according to their satisfaction with HPV vaccination behavior: the satisfied group and the unsatisfied group. Those who selected “I was satisfied with my HPV vaccination behavior” were classified as the satisfied group, and those who selected “I was not satisfied with my vaccination behavior” or “I could not say either way” were classified as the unsatisfied group.

### Exposure factors and potential confounding factors

Factors obtained via a self-administered questionnaire included the following: age, university department (nursing or non-nursing), HPV vaccination behavior (vaccinated or non-vaccinated), and number of vaccinations (once, twice, or thrice).

Regarding HPV vaccination behavior, study participants were classified into a vaccinated group and a non-vaccinated group. Those who selected “I received HPV vaccination at least once” were classified as the vaccinated group, and those who selected “I have never been HPV vaccinated” were classified as the non-vaccinated group.

In terms of knowledge of cervical cancer, HPV, and HPV vaccination, participants were asked a total of 20 items concerning cervical cancer, HPV, and HPV vaccination. The respondents were asked whether they knew or did not know about each item, and the number of items they knew was used as their knowledge score (range: 0-20).

Attitudes toward HPV vaccination were measured using the attitudes scale for HPV vaccinations ^(13)^. This scale consists of 29 items divided into eight subscales. Items were scored on a five-point Likert scale ranging from 1 (strongly disagree) to 5 (strongly agree), with higher scores indicating higher attitudes toward each sub-scale. The subscales were (1) family health awareness, (2) contact with vaccination topics, (3) ability to adjust to vaccination, (4) threat of cervical cancer, (5) high affirmation and interest regarding vaccination, (6) negative attitude/difficulty regarding vaccination, (7) anxiety about vaccines, and (8) barriers to vaccination time and cost. The reliability and validity of this scale have been previously confirmed ^(14)^.

Regarding perceptions related to sexuality, the following three items were asked. The VAS (visual analog scale) was used to ask about the possibility of getting pregnant via sexual intercourse, the possibility of contracting STIs via sexual intercourse, and the possibility of contracting cervical cancer via sexual intercourse. Participants were also asked about their sexual intercourse experience (presence or absence).

Moreover, participants were asked to respond (yes/no) whether each of the following was an influencing factor for their HPV vaccination behavior: parental recommendation, healthcare provider’s recommendation, schoolteacher’s recommendation, sexual education, vaccination of friends, cost of vaccination, problems with adverse reactions, and free coupon.

### Statistical analysis

Differences in age, university department, knowledge, attitude, and perceptions and behaviors related to sexuality between the groups satisfied and unsatisfied with their HPV vaccination behavior were determined via *t*-tests for continuous data of normal distribution. Dichotomous and categorical data were analyzed using the chi-squared test and are shown as percentages.

Logistic regression models were used to estimate the odds ratios (ORs) and 95% confidence intervals (CIs) of each factor (response variable: 1 = satisfied group, 0 = unsatisfied group). Moreover, the multivariable-adjusted ORs and 95% CIs of each factor were calculated in a model including age (continuous variable), HPV vaccination behavior (vaccinated or unvaccinated), university department (nursing or non-nursing), knowledge scores (continuous variable), attitude of contact with vaccination topics (scores ≥9 or <9), anxiety about vaccines (scores ≥10 or <10), and recognition of possibility of contracting STIs via sexual intercourse (continuous variable: VAS score). The subscales of attitude scale scores were delimited by each median.

Additional analysis of factors that were related to HPV vaccination behaviors was carried out. Differences in age, university department, knowledge, attitude, influence factors, and perceptions and behaviors related to sexuality between the vaccinated and non-vaccinated group with HPV vaccination among Japanese university female students were determined using *t*-tests for continuous data of normal distribution. Dichotomous and categorical data were analyzed using the chi-squared test and are shown as percentages.

Logistic regression models were used to estimate the OR and 95% CI of each factor (response variable: 1 = vaccinated group, 0 = unvaccinated group). Moreover, the multivariable-adjusted ORs and 95% CIs of each factor were calculated in a model including age (continuous variable), university department (nursing or non-nursing), knowledge scores (continuous variable), attitude of family health awareness (scores ≥16 or <16), contact with vaccination topics (scores ≥9 or <9), ability to adjust to vaccination (scores ≥16 or <16), high affirmation and interest regarding vaccination (scores ≥14 or <14), anxiety about vaccines (scores ≥10 or <10), barriers to vaccination time and cost (scores ≥10 or <10), and influence factors of parents (yes or no), healthcare provider (yes or no), school teacher (yes or no), problems with adverse reactions (yes or no), free coupon (yes or no), and sexual intercourse experience (presence or absence). The subscales of attitude scale scores were delimited by each median. All data were analyzed using SPSS statistical software (version 27; SPSS Inc, Chicago, IL, USA). All reported *p*-values were two-tailed, and values of <0.05 were considered statistically significant.

## Results

Of the 252 female university students, 102 (40.5%) selected “I was satisfied with my HPV vaccination behavior” (satisfied group), 126 (50.0%) selected “I could not say either way,” and 24 (9.5%) selected “I was not satisfied with my vaccination behavior” (unsatisfied group).

The differences in age, university department, knowledge, attitude scale, and perceptions and behaviors related to sexuality between the participants who were satisfied and unsatisfied with their HPV vaccination behavior are shown in Table 1. Of the 252 participants, the mean age was 21.3 years, 46.2% were nursing students, and 42.5% had been vaccinated. Participants who were satisfied with their HPV vaccination behavior included a significantly higher proportion of nursing students (p < 0.001), HPV vaccinated (p < 0.001), attitude of contact with vaccination topics score above the median (p = 0.001), and attitude of anxiety about vaccines below the median (p < 0.001) in comparison to the unsatisfied group. Furthermore, satisfied participants had a significantly higher age (p < 0.001) and knowledge score (p < 0.001) and significantly lower scores of recognition of the possibility of contracting STIs by sexual intercourse (p < 0.049). There were no statistically significant differences in other perceptions and behaviors related to sexuality between the satisfied group and the unsatisfied group.

**Table 1.** Differences in Characteristics between the Groups of Female Japanese University Students Satisfied and Unsatisfied with Their HPV Vaccination Behavior.

Factors	All	Satisfied group	Unsatisfied group	*p*-value
n	252	102	150	
Age, years *	21.28 ± 0.84	21.60 ± 0.85	21.07 ± 0.76	<0.001
Age: 20, %	17.5	10.8	22.0	<0.001
21, %	43.7	30.4	52.7	
22, %	32.5	48.0	22.0	
23, %	6.0	9.8	3.3	
24, %	0.4	1.0	0.0	
University Department: nursing, %	46.2	60.8	39.2	<0.001
HPV vaccination: vaccinated, %	42.5	70.6	29.4	<0.001
Number of vaccinations: three times, %	45.0	59.1	17.6	<0.001
twice, %	34.0	24.2	52.9	
once, %	21.0	16.7	29.4	
Knowledge, scores *	9.07 ± 4.99	10.97 ± 5.22	7.78 ± 4.40	<0.001
Attitude scale scores
Family health awareness ≥16^ a^, %	60.7	66.7	56.7	0.111
Contact with vaccination topics ≥9^ a^, %	49.6	61.8	41.3	0.001
Ability to adjust to vaccination ≥16^ a^, %	56.0	56.9	55.3	0.810
Threat of cervical cancer ≥10^ a^, %	71.4	71.6	71.3	0.968
High affirmation and interest regarding vaccination ≥14^ a^, %	61.1	65.3	58.0	0.219
Negative attitude/difficulty regarding vaccination ≥9^ a^, %	53.2	46.1	58.0	0.063
Anxiety about vaccines ≥10^ a^, %	57.1	44.1	66.0	<0.001
Barriers to vaccination time and cost ≥10^ a^, %	51.6	47.1	54.7	0.236
Perceptions and behaviors related to sexuality
Sexual intercourse experience: presence, %	49.6	53.9	46.7	0.258
Possibility of getting pregnant by sexual intercourse; VAS scores *^ b^	56.0 ± 20.7	54.0 ± 21.5	57.4 ± 20.2	0.198
Possibility of contracting STIs by sexual intercourse; VAS scores *^ b^	54.1 ± 20.8	50.9 ± 20.0	56.2 ± 21.2	0.049
Possibility of contracting cervical cancer by sexual intercourse; VAS scores *^ b^	44.6 ± 19.8	42.4 ± 19.7	46.2 ± 19.8	0.130

Dichotomous and categorical data were analyzed using the chi-squared or Fisher’s exact test and were shown as %. *Continuous data of normal distribution were analyzed by t-test and are shown as mean ± standard deviation. ^a^ Subscales of attitude scale scores were categorized by each median. ^b^ The scores of perceptions of possibility of getting pregnant, STIs, cervical cancer by sexual intercourse were evaluated by visual analog scale (VAS). Abbreviations: HPV; human papillomavirus, STIs; sexually transmitted infections.

The univariate and multivariable-adjusted ORs and 95% CIs for the group satisfied with their vaccination behavior are shown in Table 2. In the univariate analysis, ORs and 95% CIs for the satisfied group were 2.30 (1.63-3.24) for age (per 1 year), 7.89 (4.46-13.94) for HPV vaccinated, 2.60 (1.55-4.36) for nursing department, 1.15 (1.09-1.21) for knowledge scores (per 1 point), 2.29 (1.37-3.84) for attitude of contact with vaccination topics above the median, 0.41 (0.24-0.68) for attitude of anxiety about vaccines above the median, and 0.88 (0.78-1.000) for recognition of possibility of contracting STIs by sexual intercourse (per 1 point). After adjustment for age, HPV vaccination behavior (vaccinated or non-vaccinated), university department (nursing or non-nursing), knowledge scores, attitude of contact with vaccination topics (scores ≥9 or <9), attitude of anxiety about vaccines (scores ≥10 or <10), and recognition of possibility of contracting STIs by sexual intercourse (VAS score), the multivariable-adjusted ORs and 95% CIs for the satisfied group were 5.46 (2.51-11.89) for those with vaccinated behaviors (vs. non-vaccinated), 1.09 (101-1.17) for knowledge scores (per 1point), and 0.83 (0.72-0.96) for recognitions of possibility of contracting STIs via sexual intercourse (per 1 point).

**Table 2.** Factors Related to Satisfaction with HPV Vaccination Behavior among Female Japanese University Students.

Factors	Comparison	OR (95% CI) for satisfied group	*p*-value	Multivariable-adjusted* OR (95% CI) for satisfied group *	*p*-value
Age: years	vs. per 1year	2.30 (1.63-3.24)	<0.001	1.44 (0.96-2.15)	0.078
HPV vaccination: vaccinated	vs. non-vaccinated	7.89 (4.46-13.94)	<0.001	5.46 (2.51-11.89)	<0.001
University Department: nursing	vs. non-nursing	2.60 (1.55-4.36)	<0.001	1.24 (0.61-2.53)	0.557
Knowledge: scores	vs. per 1 point	1.15 (1.09-1.21)	<0.001	1.09 (1.01-1.17)	0.033
Attitude scale scores					
Family health awareness: ≥16^ a^	vs. <16^ a^	1.53 (0.91-2.58)	0.111
Contact with vaccination topics: ≥9^ a^	vs. <9^ a^	2.29 (1.37-3.84)	0.002	1.42 (0.69-2.94)	0.345
Ability to adjust to vaccination: ≥16^ a^	vs. <16^ a^	1.06（0.64-1.77）	0.810
Threat of cervical cancer: ≥10^ a^	vs. <10^ a^	0.99（0.57-1.73）	0.968
High affirmation and interest regarding vaccination: ≥14^ a^	vs. <14^ a^	1.39 (0.82-2.34)	0.220
Negative attitude/difficulty regarding vaccination: ≥9^ a^	vs. <9^ a^	0.62 (0.37-1.03)	0.063
Anxiety about vaccines: ≥10^ a^	vs. <10^ a^	0.41 (0.24-0.68)	<0.001	0.63 (0.34-1.18)	0.149
Barriers to vaccination time and cost: ≥10^ a^	vs. <10^ a^	0.74 (0.45-1.22)	0.236
Perceptions and behaviors related to sexuality					
Sexual intercourse experience: presence	vs. absence	1.34 (0.81-2.22)	0.259
Possibility of getting pregnant by sexual intercourse; VAS scores ^b^	vs. per 1 point	0.92 (0.82-1.04)	0.198
Possibility of contracting STIs by sexual intercourse; VAS scores ^b^	vs. per 1 point	0.88 (0.78-1.000)	0.050	0.83 (0.72-0.96)	0.012
Possibility of contracting cervical cancer by sexual intercourse; VAS scores ^b^	vs. per 1 point	0.91 (0.78-1.03)	0.131

Logistic regression analysis was used to estimate the adjusted ORs and 95% CIs for satisfaction with HPV vaccination behavior (response variable: 1 = satisfied group, 0 = unsatisfied group). *Age (continuous variable), HPV vaccination behavior (vaccinated or non-vaccinated), university department (nursing or non-nursing), knowledge scores (continuous variable), attitude of contact with vaccination topics (scores ≥9 or <9), attitude of anxiety about vaccines (scores ≥10 or <10), perceptions of possibility of contracting STIs by sexual intercourse (continuous variable: VAS score) were included in this model. ^a^ Subscales of attitude scale scores were categorized by each median. ^b^ The scores of perceptions of possibility of getting pregnant, STIs, cervical cancer by sexual intercourse were evaluated by visual analog scale (VAS). Abbreviations: HPV; human papillomavirus, STIs; sexually transmitted infections, OR; odds ratio, CI; confidence interval.

Table 3 shows the differences in age, university department, knowledge, attitude scale, influence factors, and perceptions and behaviors related to sexuality between the vaccinated group and the non-vaccinated group. Participants who were vaccinated included a significantly higher proportion of nursing students (p < 0.001), above-median scores for attitude of family health awareness (p = 0.004), attitude of contact with vaccination topics (p < 0.001), attitude of ability to adjust to vaccination (p = 0.004), attitude of high affirmation and interest regarding vaccination (p < 0.001), and below-median scores for attitude of negative attitude/difficulty regarding vaccination (p < 0.001), attitude of anxiety about vaccines (p < 0.001), and attitude of barriers to vaccination time and cost (p < 0.001). In terms of influencing factors, vaccinated participants reported higher influence of parental recommendation (p < 0.001), healthcare provider’s recommendation (p = 0.044), schoolteacher’s recommendation (p < 0.001), and free coupon (p < 0.001), and lower influencing factors for problems with adverse reactions (p < 0.001). They also reported a higher proportion of sexual intercourse experience (p = 0.011) and had significantly higher age (p < 0.001) and knowledge score (p < 0.001) than the non-vaccinated group. Perceptions related to sexuality between the vaccinated group and the non-vaccinated group were not significantly different.

**Table 3.** Differences in Characteristics and Factors Influencing HPV Vaccination Decisions between the Vaccinated Group and the Non-Vaccinated Group among Female Japanese University Students.

Factors	All	Vaccinated group	Non-vaccinated group	*p*-value
n		107	145	
Age, years *		21.70 ± 0.76	20.97 ± 0.75	<0.001
Age: 20, %		4.7	26.9	<0.001
21, %		32.7	51.7	
22, %		51.4	18.6	
23, %		10.3	2.8	
24, %		0.9	0.0	
University Department: nursing, %		62.6	35.2	<0.001
Knowledge, scores *		11.31 ± 4.74	7.42 ± 4.52	<0.001
Attitude scale scores				
Family health awareness ≥16^ a^, %		71.0	53.1	0.004
Contact with vaccination topics ≥9^ a^, %		79.4	27.6	<0.001
Ability to adjust to vaccination ≥16^ a^, %		67.3	47.6	0.002
Threat of cervical cancer ≥10^ a^, %		71.0	71.7	0.904
High affirmation and interest regarding vaccination ≥14^ a^, %		72.9	52.4	<0.001
Negative attitude/difficulty regarding vaccination ≥9^ a^, %		35.5	66.2	<0.001
Anxiety about vaccines ≥10^ a^, %		41.1	69.0	<0.001
Barriers to vaccination time and cost ≥10^ a^, %		36.4	62.8	<0.001
Influencing factors				
Parental recommendation: yes, %	46.8	85.0	18.6	<0.001
Health care provider’s recommendation: yes, %	2.8	5.6	0.7	0.044
Schoolteacher’s recommendation: yes, %	8.3	16.8	2.1	<0.001
Sexual education: yes, %	4.8	6.5	3.4	0.254
Vaccination of friends: yes, %	8.3	12.1	5.5	0.060
Cost of vaccination: yes, %	27.4	21.5	31.7	0.072
Problems with adverse reactions: yes, %	29.4	5.6	46.9	<0.001
Free coupon: yes, %	13.5	28.0	2.8	<0.001
Perceptions and behaviors related to sexuality				
Sexual intercourse experience: presence, %	58.9	42.8	0.011
Possibility of getting pregnant by sexual intercourse; VAS scores *^ b^	56.3 ± 20.9	55.8 ± 20.7	0.832
Possibility of contracting STIs by sexual intercourse; VAS scores *^ b^	54.3 ± 20.0	53.9 ± 21.5	0.880
Possibility of contracting cervical cancer by sexual intercourse; VAS scores *^ b^	45.1 ± 20.3	44.4 ± 19.5	0.780

Dichotomous and categorical data were analyzed using the chi-squared or Fisher’s exact test or Fisher’s exact tests and were shown as %. *Continuous data of normal distribution were analyzed by t-test and are shown as mean ± standard deviation. ^a^ Subscales of attitude scale scores were categorized by each median. ^b^ The scores of perceptions of possibility of getting pregnant, STIs, cervical cancer by sexual intercourse were evaluated by visual analog scale (VAS). Abbreviations: HPV; human papillomavirus, STIs; sexually transmitted infections.

The univariate and multivariable-adjusted ORs and 95% CIs for factors related to vaccination behavior for the vaccinated group are shown in Table 4. After adjustment for confounding factors, the multivariable-adjusted ORs and 95% CIs for the vaccinated group were 2.36 (1.12-5.00) for age (per 1 year), 3.45 (1.11-10.72) for above-median attitude of contact with vaccination topics, 31.86 (9.81-103.47) for influencing factors for parental recommendation, 0.06 (0.01-0.24) for influencing factors for problems with adverse reactions, and 4.96 (1.11-22.16) for influencing factors for free coupon.

**Table 4.** Factors Related to HPV Vaccination Behavior among Female Japanese University Students.

Factors	Comparison	OR (95% CI) for vaccinated group	*p*-value	Multivariable-adjusted* OR (95% CI) for the vaccinated group	*p*-value
Age: years	vs. per 1year	3.51 (2.37-5.20)	<0.001	2.36 (1.12-5.00)	0.025
University Department: nursing	vs. non-nursing	3.09 (1.84-5.19)	<0.001	1.65 (0.50-5.49)	0.414
Knowledge: scores	vs. per 1 point	1.91 (1.12-1.26)	<0.001	1.13 (0.99-1.28)	0.064
Attitude scale scores					
Family health awareness: ≥16^ a^	vs. <16^ a^	2.17 (1.27-3.68)	0.004	2.20 (0.72-6.69)	0.166
Contact with vaccination topics: ≥9^ a^	vs. <9^ a^	10.14 (5.60-18.36)	<0.001	3.45 (1.11-10.72)	0.033
Ability to adjust to vaccination: ≥16^ a^	vs. <16^ a^	2.27 (1.35-3.81)	0.002	1.28 (0.43-3.79)	0.652
Threat of cervical cancer: ≥10^ a^	vs. <10^ a^	0.97 (0.56-1.68)	0.904
High affirmation and interest regarding vaccination: ≥14^ a^	vs. <14^ a^	2.44 (1.43-4.18)	0.001	1.57 (0.49-5.04)	0.447
Negative attitude/difficulty regarding vaccination: ≥9^ a^	vs. <9^ a^	0.28 (0.17-0.48)	<0.001	0.40 (0.13-1.23)	0.108
Anxiety about vaccines: ≥10^ a^	vs. <10^ a^	0.31 (0.19-0.53)	<0.001	0.77 (0.25-2.40)	0.649
Barriers to vaccination time and cost: ≥10^ a^	vs. <10^ a^	0.34 (0.20-0.57)	<0.001	0.63 (0.21-1.88)	0.403
Influencing factors					
Parental recommendation: yes	vs. no	24.86 (12.64-48.87)	<0.001	31.86 (9.81-103.47)	<0.001
Health care provider’s recommendation	vs. no	8.55 (1.01-72.15)	0.048	1.85 (0.04-84.59)	0.752
Schoolteacher’s recommendation: yes	vs. no	9.57 (2.74-33.44)	<0.001	2.88 (0.49-16.75)	0.240
Sexual education: yes	vs. no	1.96 (0.61-6.35)	0.262
Vaccination of friends: yes	vs. no	2.37 (0.95-5.94)	0.066
Cost of vaccination: yes	vs. no	0.59 (0.33-1.05)	0.073
Problems with adverse reactions: yes	vs. no	0.07 (0.03-0.16)	<0.001	0.06 (0.01-0.24)	<0.001
Free coupon: yes	vs. no	13.73 (4.67-40.43)	<0.001	4.96 (1.11-22.16)	0.036
Perceptions and behaviors related to sexuality					
Sexual intercourse experience: presence	vs. absence	1.92 (1.16-3.18)	0.012	1.55 (0.54-4.49)	0.415
Possibility of getting pregnant by sexual intercourse; VAS scores ^b^	vs. per 1 point	1.01 (0.90-1.14)	0.831
Possibility of contracting STIs by sexual intercourse; VAS scores ^b^	vs. per 1 point	1.01 (0.90-1.14)	0.879
Possibility of contracting cervical cancer by sexual intercourse; VAS scores ^b^	vs. per 1 point	1.02 (0.90-1.16)	0.779

Logistic regression analysis was used to estimate the adjusted ORs and 95% CIs for HPV vaccination behavior (response variable: 1 = vaccinated group, 0 = non-vaccinated group). * Age (continuous variable), university department (nursing or non-nursing), knowledge scores (continuous variable), family health awareness (scores ≥16 or <16), attitude of contact with vaccination topics (scores ≥9 or <9), attitude of ability to adjust to vaccination (scores ≥16 or <16), attitude of high affirmation and interest regarding vaccination (scores ≥14 or <14), attitude of anxiety about vaccines (scores ≥10 or <10), attitude of barriers to vaccination time and cost (scores ≥10 or <10), and influencing factors of parents (yes or no), healthcare providers (yes or no), school teachers (yes or no), problems with adverse reactions (yes or no), free coupon (yes or no), as well as sexual intercourse experience (presence or absence) were included in the multivariable-adjusted model. a Subscales of attitude scale scores were categorized by each median. b The scores of perceptions of possibility of getting pregnant, STIs, cervical cancer by sexual intercourse were evaluated by visual analog scale (VAS). Abbreviations: HPV; human papillomavirus, STIs; sexually transmitted infections, OR; odds ratio, CI; confidence interval.

## Discussion

This study clearly shows the factors that are related to satisfaction with decision-making regarding HPV vaccination behavior among female university students in Japan. After adjustment for confounding factors, being vaccinated, having higher knowledge scores, and having lower awareness about the risk of STIs were associated with satisfaction with their decision-making in terms of HPV vaccination. Moreover, HPV vaccination behaviors were largely associated with parental recommendations and problems with adverse reaction issues in this population.

Making a well-informed decision that is consistent with personal values is important ^(7)^. In Japan, the need to make appropriate decisions and behavioral choices according to scientific thinking and correct judgment on health-related issues is discussed in school education curricula ^(15)^. Subject-centered decision-making must presuppose an understanding of the benefits and disadvantages of individual options and allow for free judgment based on one’s own values. Because HPV is transmitted through sexual behavior, decision-making regarding HPV vaccination requires a perspective on sexuality and the provision of correct information. Nevertheless, considering that the recommended target population for HPV vaccination is students from the sixth grade of elementary school students to the first grade of high school, it is necessary to target not only the women for intervention but also their parents.

In our study, the rate of satisfaction with decision-making was higher in the vaccinated group, in the group with higher knowledge of HPV and HPV vaccines, and among those with lower recognition of the possibility of contracting STIs via sexual intercourse. The other perceptions and behaviors related to sexuality were not associated with satisfaction with their decision-making regarding HPV vaccination. Nevertheless, parents of the target women may not want to directly address their daughters’ sexual behavior and are likely to have negative feelings about it.

Previous studies have reported that parents’ HPV vaccination decision-making can be influenced by healthcare providers ^(16)^, and parent satisfaction with provider communication may play an important role in this process. Hence, healthcare providers that offer correct information to mothers and target women will effectively influence decision-making. Moreover, HPV vaccination status was not significantly associated with an increased likelihood of sexual activity, decreased age of sexual debut, or an increased number of sexual partners ^(10)^, and the understanding of the roles of sexual behaviors in HPV transmission requires proper education ^(17)^. Moreover, in our study, satisfaction with decision-making regarding HPV vaccination and HPV vaccination status was not significantly associated with sexual intercourse experience. Therefore, concerns regarding the influence of the HPV vaccine on sexual behavior are likely unfounded for both men and women. Healthcare providers should use the results of these studies to provide correct information to parents and target women, including the perspective of sexuality.

Globally, HPV vaccination is recommended for women aged 9-14 years ^(1)^, when most have not initiated sexual activity. However, women of this age may not make their own HPV vaccination decisions, especially those that consider perspectives that are related to sexuality. Furthermore, parental consent is required for HPV vaccination for minors in Japan. Previous studies have revealed a relationship between vaccination and parental influence ^(9), (18)^. In our study, parental recommendation was a major factor that influences HPV vaccination, and problems with adverse reactions and free coupons also influenced HPV vaccination behaviors. Previous studies reported included the cost of vaccines, lack of knowledge, and perception that they were at low risk as reasons for not being vaccinated ^(19)^, similar to the results of our study. Higher levels of knowledge of cervical cancer, HPV, the HPV vaccine, and STIs influence HPV vaccination behavior ^(20), (21)^. Additionally, many of those who refused HPV vaccination doubted the safety and efficacy of the new vaccine or perceived themselves as not at risk of HPV infection ^(20)^. These findings suggest that providing information and knowledge regarding the etiology of cervical cancer and its link to HPV is an essential component of enhancing HPV vaccination behaviors.

Although vaccination recommendations from healthcare providers are a very important intervention, in our study, these were not associated with HPV vaccination. The Japanese government had previously suspended HPV vaccination recommendations, and healthcare providers may not have been able to explain the vaccination recommendations to eligible target women and their parents. Nevertheless, since the resumption of recommended HPV vaccinations in April 2022, it has become necessary for healthcare providers to provide high-quality information and correct knowledge. A previous study also reported that receiving quality explanations from healthcare providers and being satisfied with the explanations and communication from healthcare providers were significant influences on HPV vaccination, and recommendations from healthcare providers were also a major influencing factor ^(18)^. The results of our study indicate the importance of communicating correct information and knowledge regarding cervical cancer, HPV, HPV vaccine, and STIs to parents as well as to the target women for vaccination and decision satisfaction. It is also clear that HPV vaccination should be free.

Based on our study, HPV vaccine-related sexual education in schools, especially from healthcare providers, is an important intervention because the targets of this vaccination program are from the sixth grade of elementary school to the first grade of high school. In implementing this, healthcare providers should go to schools to provide high-quality, accurate information. Educational materials and program-based interventions for Japanese high-school students have been reported to affect short-term behavioral intentions ^(22)^, and mothers’ narrative messages may be persuasive when targeting mothers to promote HPV vaccination ^(23)^. Because HPV is transmitted through sexual behavior, as an aid to decision-making support, perspectives on sexuality should also be accurately communicated to avoid misunderstandings. Intervention with parents (especially mothers) is also necessary. To this end, parents should be allowed to observe during school sexual education, and seminars for parents should be conducted during Parent-Teacher Association (PTA) activities. Additionally, we believe that disseminating high-quality, accurate information via social networking services for women who are not eligible for the recommended vaccination would be effective. Being vaccinated, having higher knowledge scores, and having lower awareness about the risk of STIs were associated with satisfied decision-making concerning HPV vaccination. Providing younger people with correct information about cervical cancer, HPV vaccines, and STI prevention would contribute to increased satisfaction with HPV vaccination and decision-making about HPV vaccination.

To our knowledge, this study is the first to examine the factors that include perspectives on sexuality related to satisfaction regarding their decisions regarding HPV vaccination for female university students in Japan. Nevertheless, there are some study limitations. First, the cross-sectional study design precludes any conclusions about the causal nature of the observed associations. Therefore, a prospective study is required to confirm our findings. Second, data were obtained only from female university students who agreed with the study aims and fully completed the questionnaire. Therefore, it is possible that the target population of interest for our study was participating at a higher rate than the general population. This was also likely because the percentage of the vaccinated group was higher than the general vaccination rate ^(24)^ for the same age group. The questionnaire was developed by the authors and cannot be said to be sufficiently reliable and valid. Furthermore, the question about the subjects’ experience with sexual intercourse may be causing the low response rate. As such, we cannot deny the possibility of selection bias because the response rate was 15.1%. Third, other potential confounding factors that were not considered in this study included living environment and individual personality. Despite these potential limitations, the present findings support the conclusion that providing younger people with correct information about cervical cancer, HPV vaccines, and STI prevention is important in promoting improved decision-making concerning HPV vaccination among female university students in Japan.

## Article Information

### Conflicts of Interest

None

### Sources of Funding

This work was supported by the Fund for Pfizer Health Research Foundation.

### Acknowledgement

We are grateful to all participants who participated in this research. We thank Tomoko Hina, Junichi Asada, and Daigo Hayashi for their advice on this research. We also thank John Daniel from Edanz (https://jp.edanz.com/ac) for editing a draft of this manuscript.

### Author Contributions

Chie Koh contributed to the conception and design of this study; collection, analysis, and interpretation of data; funding acquisition; project administration; and writing the original draft.

Minako Saho, Yukari Nakajima, Miho Furuyama, and Kanako Yamada contributed to project participation and review. Kaori Watanabe and Yuichirou Nakai contributed to the review and supervised the whole study process. All the authors read the final manuscript and approved the submission of the article.

### Approval by Institutional Review Board (IRB)

Institutional Review Boards of Osaka Prefecture University (Approval code. 2020-51)

## References

[1] World Health Organization (WHO). Cervical cancer [Internet]. [cited 2022 Feb 22]. Available from: https://www.who.int/news-room/fact-sheets/detail/cervical-cancer.

[2] Centers for Disease Control and Prevention (CDC). Basic information about cervical cancer [Internet]. [cited 2021 Dec 14]. Available from: https://www.cdc.gov/cancer/cervical/basic_info/index.htm.

[3] Ministry of Health, Labour and Welfare, Japan. Ref1.History of and response to the HPV vaccine [Internet]. [cited 2015 Oct 22]. Available from: https://www.mhlw.go.jp/file/05-Shingikai-10901000-Kenkoukyoku-Soumuka/0000103090.pdf. Japanese.

[4] Ministry of Health, Labour and Welfare, Japan. HPV vaccination from April 2022 [Internet]. [cited 2022 Apr 1]. Available from: https://www.mhlw.go.jp/content/10906000/000911549.pdf. Japanese.

[5] Sekine M, Kudo R, Yamaguchi M, et al. Japan’s ongoing crisis on HPV vaccination. Vaccines. 2020;8(3):362. 32640691 10.3390/vaccines8030362 PMC7565470

[6] Suzuki Y, Sukegawa A, Ueda Y, et al. Effect of a brief web-based educational intervention on willingness to consider human papillomavirus vaccination for children in Japan: randomized controlled trial. J Med Internet Res. 2021;23(9):e28355. 34569941 10.2196/28355 PMC8506261

[7] O’Connor AM. Validation of a decisional conflict scale. Med Decis Making. 1995;15(1):25-30. 7898294 10.1177/0272989X9501500105

[8] Harper DM, Irons BB, Alexander NM, et al. Quantifying the decisional satisfaction to accept or reject the human papillomavirus (HPV) vaccine: a preference for cervical cancer prevention. PLOS ONE. 2014;9(2):e88493. 24551110 10.1371/journal.pone.0088493 PMC3925140

[9] Wanderley MS, Sobral DT, Levino LA, et al. Students’ HPV vaccination rates are associated with demographics, sexuality, and source of advice but not level of study in medical school. Inst Med Trop Sao Paulo. 2019;61:e70. 10.1590/S1678-9946201961070 PMC6922017 31859847

[10] Brouwer AF, Delinger RL, Eisenberg MC, et al. HPV vaccination has not increased sexual activity or accelerated sexual debut in a college-aged cohort of men and women. BMC Public Health. 2019;19(1):821. 31238911 10.1186/s12889-019-7134-1 PMC6593582

[11] The Japanese Association for Sex Education. White paper on “sexuality of youth” report on the 8th national survey on sexual behavior of youth. Tokyo: Shogakukan; 2019. 9-24 p. Japanese.

[12] Kobayashi Y, Asakura T. Factors influencing female high-school students’ intention to receive the human papilloma virus vaccine: an investigation using the health belief model. J Educ Res. 2015;31:13-26. Japanese.

[13] Kobayashi Y. Psychosocial factors related to cervical cancer prevention behavior among female high-school students. Tokyo: KazamaShobo; 2016. 72-3 p. Japanese.

[14] Kobayashi Y, Asakura T. Psychosocial factors related to receive the human papilloma virus vaccine among female high-school students: a path analysis based on modified Health Belief Model. Indic Welfare. 2015;62(11):15-24. Japanese.

[15] Yamamoto N. The junior high school health and physical education to cultivate zest for life-focusing on decision-making process in health education. Bulletin of Fukuoka University of Education Graduate School of Education Division of Professional Practice in Education. 2015;5:135-42. Japanese.

[16] Kornides ML, Fontenot HB, McRee AL, et al. Associations between parents’ satisfaction with provider communication and HPV vaccination behaviors. Vaccine. 2018;36(19):2637-42. 29627236 10.1016/j.vaccine.2018.03.060 PMC5915964

[17] Zou H, Wang W, Ma Y, et al. How university students view human papillomavirus (HPV) vaccination: a cross-sectional study in Jinan, China. Hum Vaccin Immunother. 2016;12(1):39-46. 26308701 10.1080/21645515.2015.1072667 PMC4962712

[18] Kornides ML, McRee AL, Gilkey MB. Parents who decline HPV vaccination: who later accepts and why? Acad Pediatr. 2018;18(2S):S37-43. 29502636 10.1016/j.acap.2017.06.008 PMC5859546

[19] Ratanasiripong NT, Sri-Umporn S, Kathalae D, et al. Human papillomavirus (HPV) vaccination and factors related to intention to obtain the vaccine among young college women in Thailand. J Health Res. 2018;32(2):142-51.

[20] Wong LP, Sam IC. Ethnically diverse female university students’ knowledge and attitudes toward human papillomavirus (HPV), HPV vaccination and cervical cancer. Eur J Obstet Gynecol Reprod Biol. 2010;148(1):90-5. 19910102 10.1016/j.ejogrb.2009.10.002

[21] Leung JTC, Law CK. Revisiting knowledge, attitudes and practice (KAP) on human papillomavirus (HPV) vaccination among female university students in Hong Kong. Hum Vaccin Immunother. 2018;14(4):924-30. 29232166 10.1080/21645515.2017.1415685 PMC5893186

[22] Shida J. Short-term efficacy of the educational program to enhance behavioral intentions to prevent cervical cancer among Japanese female high school students. J Sch Health. 2019;15:11-24.

[23] Okuhara T, Ishikawa H, Okada M, et al. Persuasiveness of statistics and patients’ and mothers’ narratives in human papillomavirus vaccine recommendation messages: a randomized controlled study in Japan. Front Public Health. 2018;6:105. 29707533 10.3389/fpubh.2018.00105 PMC5906532

[24] Ministry of Health, Labour and Welfare, Japan. Immunization information. Number of routine immunizations performed. HPV vaccination from 2013 to 2021 [Internet]. [cited 2023 Jul 24]. Available from: https://www.mhlw.go.jp/topics/bcg/other/5.html.

